# General Sense of Perceived Self-Efficacy and Loneliness Among Polish Adolescents: Communication with Peers as Mediator

**DOI:** 10.3390/brainsci15090946

**Published:** 2025-08-29

**Authors:** Małgorzata Szcześniak, Agata Hiacynta Świątek, Aniela Szczerba, Karolina Szpunar, Adam Falewicz

**Affiliations:** 1 Institute of Psychology, University of Szczecin, 71-017 Szczecin, Poland; agata.swiatek@usz.edu.pl (A.H.Ś.); adam.falewicz@usz.edu.pl (A.F.); 2 XII Liceum Ogólnokształcące im. C. K. Norwida, 31-941 Kraków, Poland; anielkaszczerba@gmail.com; 3 VI Liceum Ogólnokształcące im. Stefana Czarnieckiego, 70-382 Szczecin, Poland; karolina.szpunar09@gmail.com

**Keywords:** self-efficacy, loneliness, adolescents, communication with peers, openness in communication, difficulty in communication

## Abstract

**Background**: Adolescence is a particularly vulnerable period for experiencing loneliness. According to the latest statistics, there are worldwide increases in adolescents’ social isolation caused by different psychosocial challenges. A number of different studies have linked a strong belief in being successful at doing something to lower levels of feeling lonely. **Objectives**: Because we know less about the potential mediators of this relationship, the aim of the current study was to assess: (1) the association between self-efficacy and loneliness; (2) the mediatory role of peer communication. **Methods**: A total of 191 primary and high school students (M_age_ = 16.22; SD = 1.44) completed the General Self-Efficacy Scale, the De Jong Gierveld Loneliness Scale, and the Scale of Communication of Adolescents with Peers. **Results**: The results of the correlation analyses revealed that self-efficacy was negatively associated with loneliness and difficulty in adolescents’ communication with peers. Conversely, self-efficacy was positively associated with openness in adolescents’ communication with peers. Moreover, the outcomes of the PROCESS macro for SPSS (model number 4; Hayes, 2013) showed that openness in adolescents’ communication with peers and difficulty in this communication were mediators in this relationship. **Conclusions**: Young people who strongly believe in their abilities to achieve success may be more willing to share ideas and personal experiences with others and have less difficulty in communicating with peers, which may lead to a reduced sense of loneliness.

## 1. Introduction

Adolescence is a particularly vulnerable period for experiencing loneliness [1], starting from hypervigilance about social threats to changes in social and emotional functioning (becoming independent from parents, searching for belonging among peers) [2]. Buecker et al. [3] identified many risk factors that increase loneliness in teenagers (e.g., low quality of social relationships, low peer acceptance, low self-esteem, social anxiety, peer victimization, shyness, depression, and neuroticism). According to the latest statistics [4], there are worldwide increases in adolescents’ social isolation caused by different psychosocial challenges. Twenge et al. [4] found that loneliness is particularly prevalent among teenage girls, and school loneliness is pronounced where there is high Internet and smartphone access.

Loneliness, although normative for adolescents, is associated with poor physical and mental health [5]. Although it is known that young people use various beneficial strategies to cope with the feeling of loneliness [6], previous findings encourage the search for protective factors against severe loneliness in adolescents. In our study, we focus on peer communication skills as potentially the most direct way to overcome social isolation. A number of different studies have linked a strong belief in being successful in doing something [7] to lower levels of feeling lonely [8,9]. Hence, we would like to explore self-efficacy beliefs in the context of communication with peers and the feeling of loneliness.

### 1.1. Loneliness

Loneliness is defined as perceived social isolation, understood as the absence of social relationships or their low quality, affecting people of different ages [10,11]. Thus, the feeling of loneliness may persist despite the physical presence of other people. In a study conducted by Igami and colleagues [12], involving over 248,000 adolescents from a total of 70 countries, the overall prevalence of loneliness was 11.7% of respondents. However, there are differences in the severity of loneliness across countries—the highest rates are found in Africa and the Eastern Mediterranean. Research conducted in Tanzania revealed that 17.4% of respondents felt significant loneliness [13], which only confirms the above data. Studies measuring loneliness rates during the COVID-19 pandemic have shown that approximately half of adolescents and children experience at least moderate loneliness and a significant average increase in loneliness compared to pre-pandemic levels [14].

In research based on adolescents, Houghton et al. presented a model of loneliness consisting of four interrelated factors: friendship, perceived isolation, positive attitudes toward loneliness, and negative attitudes toward loneliness [15]. Mahon et al. [16] found the following predictors of loneliness in adolescence: sex, shyness, self-esteem, depression, self-disclosure, stress, and age. Furthermore, Japanese researchers [17] indicated that parental psychological stress and victimization among 10-year-old children were independent factors predicting increased and chronic loneliness in the first half of adolescence. Thakur et al. [18] showed that adolescents who exhibited symptoms of depression and aggression before the COVID-19 experienced pandemic loneliness earlier. A tendency to anxiety preceded the onset of mid-pandemic loneliness. Additionally, loneliness during the pandemic was more prevalent among young women reporting previously experienced trauma.

There is also a body of research that shows the negative effects of loneliness on teenagers [19,20,21]. Christiansen et al. [5] wrote that feeling lonely was associated with an increased risk of diseases such as joint diseases, asthma, migraines, long-term mental illness, alcohol abuse, and hypertension. Another study confirmed not only the association of loneliness with physical ailments (such as stomach pain) but also with sleep disorders, difficulty falling asleep, and feeling tired upon waking [22]. Chronic loneliness and loneliness that increases over time co-occur with depressive symptoms and more frequent suicide attempts [23]. Moreover, loneliness promotes bad health habits, which means that it leads to decreased or a lack of physical activity [24].

In recent years, we have observed changes in the way adolescents interact with others, increasing the use of technology for various purposes. As Guazzini et al. [25] show, feelings of loneliness have increased during the pandemic, but young people maintain a sense of community. At the same time, the pandemic may have made people more accustomed to loneliness or deepened their isolation. A study conducted on an Italian sample [26] revealed three profiles of social self-isolation, including a doubling of the number of people who never meet with their friends after the pandemic.

### 1.2. Self-Efficacy

Díaz-Mujica et al. [27] have been writing about the growing importance of the psychological concept of self-efficacy in research since 1997, and referring to Bandura [28], identify it as beliefs about oneself regarding one’s own coping abilities. As Schwoerer [29] (p. 113) explains, “While self-efficacy itself is inherently task specific, the specificity of self-efficacy with respect to a given task varies.” The literature provides information about four sources of self-efficacy: the experience of mastery, affective and physiological states, social/verbal persuasion, and vicarious experience [30]. Perceived own abilities to complete tasks and achieve goals is a salutogenic psychological factor [31] and may be considered as a buffer against everyday stress [32].

Three facets of self-efficacy were predictors for positive coping, positive orientation, and hedonic balance among Italian adolescents during the lockdown [33]. In a Peruvian study, self-efficacy was more important to teens’ mental health than their Body Mass Index [34]. The study by Indiana and Sagone [35] indicates a positive correlation between self-efficacy in life skills and functional coping strategies, well-being, and resilience in this age group. Kleppang and colleagues [31] cite research showing that self-efficacy, among other things, reduces risky behaviors in adolescents and is linked to resilience and academic achievements. Rossi et al. [36] showed that among students aged 14–19, higher self-efficacy correlates positively with internal motivation and negatively with external motivation. Self-efficacy motivates teenagers to continue engaging in their passions, correlating, for example, with intrinsic motivation to sing [37]. Loton and Waters [38] analyze papers linking self-efficacy to well-being in teenagers and cite studies indicating that self-efficacy is a predictor of life satisfaction, happiness, and lower levels of anxiety. In a Polish study among 13-year-old adolescents, self-efficacy was found to be dependent on family socioeconomic status and, acting as a mediator, positively affected quality of life [39]. Moreover, self-efficacy moderates the relationship between post-traumatic stress disorder symptoms and posttraumatic growth in a Chinese adolescent sample studied in the context of the pandemic [40].

Comparative studies across 14 countries [41] revealed significant differences in perceived self-efficacy between groups of different nationalities. In Schwarzer’s [41] study, the highest scores were achieved by participants of Spanish, Russian, and German origin, while the lowest self-efficacy was observed among Japanese, Chinese, and Polish citizens. Other studies indicate that individual levels of self-esteem change throughout the lifespan and are known to increase from the teenage years to middle adulthood, then to decrease [42]. But it can also change in the short term. The results of experimental studies on young adults indicate that changes in the level of self-efficacy are important for increasing/decreasing their engagement in tasks and studying [43]. In the study by Shahrbabaki et al. [44], increased anxiety was found to reduce self-efficacy among Iranian adolescents in the context of pandemic-related anxiety.

Self-efficacy also depends on other psychological characteristics. For example, higher resilience predicted higher self-efficacy in adolescents [45]. Peng et al. [46] also indicate that psychological resilience is a predictor of self-efficacy, which builds confidence in adolescents’ abilities.

Taking into account the previously cited works that self-efficacy is important for many areas of a person’s functioning, we assume that it is also important for verbal communication and general coping with interpersonal relationships.

### 1.3. Communication with Peers

Communication means the exchange of information, and its quality is both a measure of and a contributor to the success of interpersonal relations [47]. By accommodating to the interlocutor (pace, vocabulary, pronunciation, duration of the conversation), one can get closer to or move away from other people, which promotes satisfaction when the person feels that the interlocutor is accommodative [48]. American research among adolescents indicates 14 communication skills, the most important of which for them were related to empathy [49]. Valkenburg and Peter [50] observed that teenagers gained psychosocial autonomy by learning to present themselves to others and, based on feedback, adjusted their self-presentation. In this way, through peer communication, they developed their identity and increased their sense of belonging. Peer communication among adolescents also mediated the relationship between their self-esteem and life satisfaction [51].

Relationships with peers are a space for misunderstandings, negotiating, but also for developing empathy. Peers socialize with each other by imitating, encouraging, modeling and rewarding, and acquire social skills needed in adult life [52]. Nowadays, online communication plays an increasingly important role in contacts with others [50], and the virtual environment has become a space for teenagers to carry out developmental tasks [53]. Maintaining online contact with offline friends positively correlates with adolescents’ well-being, but intensive communication with persons known only online is negatively associated with well-being [54]. The social media environment can foster a decrease in belonging [55], so extensive engagement with it may increase loneliness. In addition, we suppose that the way of conversing online (e.g., responding in a time-delayed manner or detached from non-verbal messages) does not weaken the ability to engage in satisfactory peer communication among adolescents.

Communication style may play an important role in perceived loneliness. In their review article about loneliness, Perlman and Peplau [10] (p. 43) cite Warren Jones’ experimental studies on differences in communication between lonely and non-lonely people. They indicate that lonely individuals are non-responsive and self-focused: they give answers more slowly, ask fewer questions, and change the subject more frequently. Recent data [56] show that adolescents’ higher levels of social anxiety predict their poorer communication skills. Edwards et al. [57] note that individuals who communicate expressively (discharging their current feelings and thoughts with little regard for the conversation partner) experience higher levels of loneliness than peers with other communication styles, and individuals who communicate rhetorically are more satisfied with their social support systems than others.

### 1.4. Hypotheses

The results of the study on a group of 481 teenagers [58] show that three areas of self-efficacy (academic, emotional, and social) are negatively correlated with the feeling of loneliness, with the strongest correlation being found in social efficacy (*r* = −0.40; *p* < 0.01). Heiman and Olenik-Shemesh [59], conducting research involving adolescents with and without learning disabilities, observed a moderate negative association between self-efficacy and loneliness (*r* = 0.45, *p* < 0.001). A negative correlation between social self-efficacy and loneliness was also similarly noted by Erozkan and Denis [60], Galanaki and Kalantzi-Azizi [61], and Gazo et al. [62]. Based on the well-documented relationships between these variables, we proposed the following hypothesis:

**H1:**  *Self-efficacy correlates negatively with the feeling of loneliness.*

Going beyond the context of the age group we studied, Axboe et al. [63] report findings from the literature indicating increased effectiveness resulting from social skills training. The opposite claim, that individuals who perceive themselves as effective will demonstrate better communication quality, seems less well-documented. Self-efficacy has already been studied by other authors as a mediator of satisfaction and competence in interpersonal relationships. A study conducted by Rubin et al. [64] on 309 college students showed that higher self-efficacy predicts higher interpersonal communication competence and satisfaction with communication. Therefore, we proposed another hypothesis:

**H2:**  *A sense of self-efficacy correlates positively with openness in communication with peers (H2a) and negatively with difficulty in communication with peers (H2b).*

Loneliness can discourage contact and become chronic, as increased self-focus and concern for one’s own interests can foster avoidance of others to protect oneself from social threats and rejection [2]. Communication is hindered when a person feels the need to defend themselves and anticipate negative reactions. For example, Gaffney [65] cited Frederickson’s [66] observation that students find it difficult to verbally express their drawing ideas when they feel threatened or perceive the environment as hostile. And, as previously mentioned, the style of communication in dyads of lonely people differs from that of non-lonely people [10,57]. It is probably lower-quality communication that is more focused on the needs of the speaker than on the needs of the interlocutor. Based on these assumptions, we proposed a further hypothesis:

**H3:**  *Openness in peer communication with peers negatively correlates with loneliness (H3a), and difficulty in communication with peers positively correlates with loneliness (H3b).*

**Figure 1:**
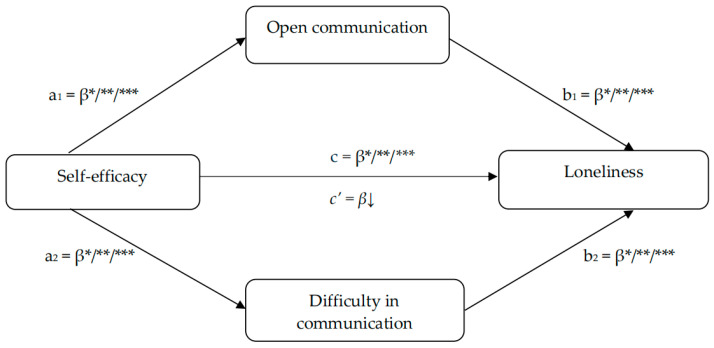
A conceptual model of the mediation of open communication and difficulty in communication in the association between self-efficacy and loneliness; *** *p* < 0.001; ** *p* < 0.01; * *p* < 0.05.

As described earlier, self-efficacy and the quality of peer communication appear to be correlated with each other, as well as with perceived loneliness. We exploratively hypothesized that general self-efficacy would be an independent variable that may positively predict higher open peer communication and lower difficulty in communication, consequently leading to reduced perceived loneliness:

**H4:**  *The openness and difficulty in adolescents’ communication mediate the relationship between perceived self-efficacy and loneliness (Figure 1).*

## 2. Materials and Methods

### 2.1. Participants and Procedure

The study involved primary and secondary school students living in three Polish provincial cities with populations exceeding 380,000; 800,000; and 2 million inhabitants (N = 191). The vast majority of them were girls (81%; 19% were boys). The mean age was M = 16.22 (SD = 1.44) and the age range was between 12 and 19. Study participants were asked about their number of real and virtual friends, and whether they had any siblings. The average number of real friends was 5 and virtual friends was 2. When asked about the number of siblings, most students declared having one sibling (*n* = 102; 53.4%), followed by those who had two (*n* = 40; 20.9%). Twelve students indicated having three or more brothers and/or sisters (6.3%) and 37 students (19.4%) indicated they were only children.

Questionnaire data were collected using a research link prepared within the Google Forms platform between 20 January and 16 February 2024. The criteria for inclusion in the study were the age of the respondents corresponding to the developmental period of adolescence, their consent to participate in the study and the consent of their parents or legal guardians. Students who did not meet the above-mentioned conditions were excluded from the study. Recruitment was carried out using convenience sampling by three of the co-authors of the article, who had access to schools in these localities. Contact was made through teachers. Parents were informed about the study at regular parent–teacher meetings. Subsequently, an informational meeting with the participants took place during weekly homeroom classes. Before sending out the link, both young people and their parents or legal guardians were familiarized with the purpose of the study and assured that it would not result in any consequences affecting the respondents. They were also informed that the study was voluntary, that it had the approval of the Bioethics Committee, and that they could discontinue it at any time, also without any consequences.

### 2.2. Scale of Communication of Adolescents with Peers

The Scale of Communication of Adolescents with Peers (SCAP), developed by Napora [47], assesses adolescents’ opinions on the positive and negative aspects of communication with peers. The SCAP contains 20 statements and consists of two subscales with 10 items for each subscale. The subscale of open communication measures adolescents’ capacity to discuss various issues such as friendship, intimate matters, school problems, or substance abuse (e.g., “I have no difficulty openly expressing all my feelings, even my deepest ones”). The subscale of difficulty in communication estimates adolescents’ lack of trust in their relationship with their peers, fear of asking something during conversation, and likelihood of shunning others by not talking to them when they express an opinion different from their own (e.g., “I don’t feel like I can express my true views on some things”). All the statements are answered on a 5-point Likert scale (1 = strongly disagree and 5 = strongly agree). The SCAP demonstrates high internal consistency. In the original study, the value of Cronbach’s alpha was 0.90 for both subscales. In the present study, the value of Cronbach’s alpha was 0.88 for openness and 0.68 for difficulty, which corresponds to previous results in other studies [39].

### 2.3. De Jong Gierveld Loneliness Scale

The De Jong Gierveld Loneliness Scale (DJGLS), developed by de Jong Gierveld and Kamphuls [67] and adapted into Polish by Humenny and Grygiel [68], is a unidimensional valid instrument that measures loneliness in its two dimensions: social (e.g., “I can call on my friends whenever I need them”) and emotional (e.g., “I experience a general sense of emptiness”). Participants rate each statement on a 5-point Likert scale ranging from 1 = strongly agree to 5 = strongly disagree. Higher scores (a minimum of 11; a maximum of 55) indicate higher levels of loneliness. The Cronbach’s alpha coefficient in the original Polish version was 0.89. Similarly, in the current study, the reliability was high with α = 0.89.

### 2.4. General Self-Efficacy Scale

The General Self-Efficacy Scale (GSES), authored by Schwarzer and Jerusalem [69] and adapted into Polish by Juczyński [70], is a brief self-report tool used to assess perceived self-efficacy and optimistic self-belief, especially in difficult and stressful life events (e.g., “I can remain calm when facing difficulties because I can rely on my coping abilities”). This unidimensional self-administered scale consists of 10 items. The participants evaluate each statement on a 4-point scale that ranges from 1 = not at all true to 4 = exactly true. The higher the final result (a minimum of 10; a maximum of 40), the stronger the general self-efficacy. Previous studies confirm very good reliability of the GSES with coefficient alphas between 0.76 and 0.90. In the present study, Cronbach’s alpha was 0.81.

All three questionnaires are publicly available and free for use, and no special permission is required.

### 2.5. Statistical Analysis

All data were measured and analyzed using the IBM SPSS Statistics software package, version 25. There was no problem of item non-response from participants since the data were gathered through an Internet platform.

Descriptive statistics in the form of measures of central tendency (means), dispersion (standard deviation), and normality of a dataset (skewness, kurtosis, and the Shapiro–Wilk test) were checked. Indices of skewness and kurtosis for self-efficacy, loneliness, and the two forms of communication with peers less than ±1 were assumed to be an approximately normal distribution. With the Shapiro–Wilk test, a significance value of *p* > 0.05 was considered a sign of normality of the data.

The strength of correlation coefficients was specified according to the values recognized in the social sciences: no association (between ±0.00 and ±0.14), weak (between ±0.15 and ±0.29), moderate (between ±0.31 and ±0.59), and a strong relationship (between ±0.60 and ±1.0) [71].

A linear regression analysis was calculated to (1) diagnose the problem of strong relationships between the explanatory variables, (2) detect outliers, and (3) assess whether sociodemographic variables would affect the relationship between perceived self-efficacy and loneliness.

In order to diagnose multicollinearity, the tolerance statistics and the variance inflation factor (VIF) were assessed. Tolerance values of 0.01 or less and a VIF larger than 10 were considered as indices of high intercorrelations between perceived self-efficacy and loneliness. A Mahalanobis distance with *p* < 0.001 and Cook’s distance higher than 1 were interpreted as signs of influential cases.

The participants’ sex, age, number of real friends, number of virtual friends, and presence or lack of siblings were examined to control for their potential confounding effects in the relationship between perceived self-efficacy and loneliness. The outcomes of previous research showing differences in self-efficacy and loneliness were the justification for choosing the above-mentioned variables as possible confounders. For example, there is empirical evidence that men/boys score higher in self-efficacy than women/girls [72,73,74]. When it comes to loneliness, the results are inconsistent. In some studies, women seem to be more adept at providing interactions that lead to avoiding loneliness [75]. In other studies, women report higher levels of loneliness than men [76]. With regard to the number of real and virtual friends, Walla et al. [77] observed via electroencephalography that the human brain distinguishes between virtual and real friends. Thus, the results obtained by researchers confirm the special role of friendship. In fact, the presence of real friends provides support and reinforces one’s value [78] and has a protective effect against loneliness [79]. In other studies, it has been found that fewer reciprocal and unilateral friendships (virtual friends are often considered as such) are associated with higher levels of loneliness among adolescents [80]. Finally, the research confirms that the mere fact of having or not having siblings influences an individual’s self-efficacy [81]. Moreover, sibling relationship quality, especially sibling warmth, was negatively linked to loneliness [82]. The above-mentioned potential confounders were entered at Step 1. Self-efficacy and communication with peers were entered at Step 2.

The PROCESS macro for SPSS in its version 3.2, proposed by Hayes [83], was used to conduct a parallel mediation analysis (Model 4) and verify the extent to which self-efficacy affects loneliness among adolescents through communication with peers. Self-efficacy served as the independent variable, and loneliness was the dependent variable. The subscale of open communication and the subscale of difficulty in communication were entered as parallel mediators (Figure 1). The mediation model was based on the bootstrapping method with 5000 replications and 95% confidence intervals (CIs). The mediating effect was considered significant if the interval did not contain 0.

In addition to the statistical significance of the indirect effects, the magnitude of the effect size (v^2^) for the model was calculated, using the SmartPLS4 software application. As suggested by Gaskin et al. [84], we assumed values of 0.175 for a large effect, 0.075 for medium, and 0.01 for small as the thresholds for v^2^.

G*Power 3.1.9.4 with a bivariate normal model correlation was computed to specify a priori the required sample size. Based on meta-analyses by Richard et al. [85], we considered a small effect size of 0.21, which is typical in social psychology, with a significance level of 0.05 and a power of 0.90. The output showed that the sample would need approximately 187 participants.

## 3. Results

### 3.1. Descriptive Statistics

A summary of the mean (M), standard deviation (SD), and measures used to assess the normality of a dataset (skewness, kurtosis, and Shapiro–Wilk) for self-efficacy, loneliness, openness, and difficulty in communication are presented in Table 1.

Although only difficulty in communication had an insignificant Shapiro–Wilk value, we assumed that the data was very close to a normal distribution because none of the variables surpassed values of skewness and kurtosis of ±1. Therefore, a Pearson correlation analysis was calculated.

### 3.2. Correlations

The Pearson analysis (Table 2) showed that the scores of self-efficacy, loneliness, openness in communication with peers, and difficulty in communication with peers significantly correlated with each other (*p* < 0.001). Consistent with the hypotheses, self-efficacy correlated negatively with loneliness (H1) and difficulty in communication with peers (H2b), and positively with openness in communication with peers (H2a). Moreover, open peer communication was negatively associated with loneliness (H3a), and difficult peer communication was positively correlated with loneliness (H3b). The observed magnitude of the correlation coefficients was between small and moderate.

### 3.3. Multicollinearity and Confounding Variables

The multiple regression computed for all variables included in the model displayed a VIF between 1.03 and 1.28, and a tolerance rate ranging from 0.700 to 0.976. Both outcomes denote the absence of a multicollinearity problem. The Mahalanobis distance showed a total of four outliers with a *p*-value lower than 0.001, thus suggesting that these four data points varied significantly from the other observations. However, Cook’s value was between 0.000 and 0.063 (lower than the cut-off of 1), thus confirming the lack of problematic cases. Moreover, since the correlational results with and without outliers did not differ substantially, we decided to leave the outliers in the dataset.

After controlling for confounding variables, we observed that the participants’ sex, age, number of real friends, number of virtual friends, and presence or lack of siblings explained 8% of the variance (*R*^2^ = 0.080). The predictors of self-efficacy, openness in communication, and difficulty in communication explained an additional 31.6% of the variance, even after adjusting for the effect of sex (*β* = −0.011, *t* = −0.191, *p* = 0.848), age (*β* = 0.009, *t* = 0.156, *p* = 0.878), number of real friends (*β* = −0.131, *t* = −2.182, *p* = 0.030), number of virtual friends (*β* = −0.031, *t* = −0.512, *p* = 0.609), and presence or lack of siblings (*β* = 0.038, *t* = 0.657, *p* = 0.512). Only the number of real friends had a significant effect, indicating that as the number of actual friends increases, the level of loneliness decreases. This result suggests that not only the mediators influenced the relationship between self-efficacy and loneliness, but also the presence of real friends.

### 3.4. Mediation Analysis

Table 3 presents the results of the PROCESS macro for SPSS on the relationship between self-efficacy and loneliness. Bootstrap estimates for the indirect effect, based on 5000 bootstrap samples, showed that zero was outside of the lower and upper bounds of the CIs in the model. More precisely, the study found that both dimensions of communication with peers (openness and difficulty) mediated the relationship between the predictor (self-efficacy) and the outcome variable (loneliness).

**Figure 2:**
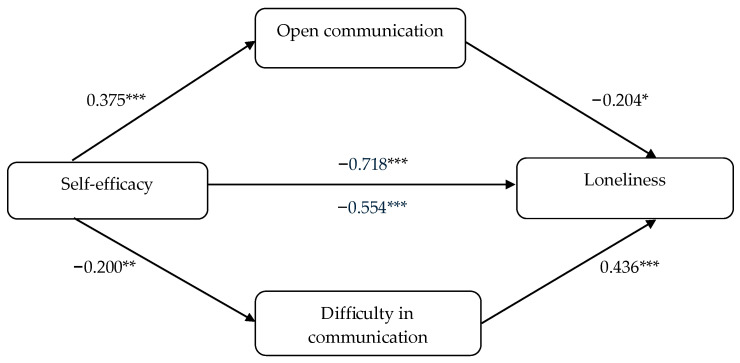
Mediatory effect of communication with peers in the relationship between self-efficacy and loneliness; *** *p* < 0.001; ** *p* < 0.01; * *p* < 0.05.

The mediation model was significant and supported by the bootstrapping CIs (Table 3) because no zero was present (thus suggesting a mediating role of both mediators on the relationship between self-efficacy and loneliness). In the case of the mediator of openness in communication with peers, its effect size (v^2^) was large v^2^ = 0.141. In contrast, the effect size of difficulty in communication was medium, with v^2^ = 0.040. Figure 2 presents graphically role of both dimensions of communication with peers in the relations between self-efficacy and loneliness.

## 4. Discussion

The purpose of our study was to examine how self-efficacy is related to loneliness and what role adolescents’ communication with peers plays in this relationship. The empirical data obtained allowed us to confirm all correlational hypotheses and demonstrate the mediating role of openness and difficulties experienced by adolescents in their relationship with their peers.

Consistent with our first hypothesis (H1), self-efficacy was negatively associated with loneliness. Previous research also confirmed that a higher sense of self-efficacy is accompanied by a lower sense of loneliness. For example, Tu and Zhang [86] demonstrated such a relationship on a group of Chinese undergraduates (at age M = 19.02; SD = 1.26, which is a slightly older group than the one presented in the current study). In their study, self-efficacy was also shown to mediate the relationship between loneliness and subjective well-being (measured as stress, depression, and life satisfaction). A study by Erozkan and Deniz [60] showed that in a group of Turkish high school students (at age M = 16.45; SD = 1.35, which is similar to our age group), social efficacy, along with learned resourcefulness, was a significant predictor of loneliness. In a study by Wei et al. [87], social self-efficacy was one of the significant predictors of loneliness in a group of freshman college students (slightly older than our respondents, with an average age of M = 18.31; SD = 0.47). Similarly, in a longitudinal study by Calandri et al. [88], emotional self-efficacy at time point 1 negatively predicted subsequent loneliness at time point 2 in a group of early adolescents (at the age of M = 13; SD = 0.3). Taken together, these studies demonstrate how the perceived ability to cope with life in general, as well as with creating and maintaining relationships, contributes to shaping perceived levels of loneliness. Similarly (although in a group slightly older than ours), a negative relationship between social self-efficacy and loneliness was shown in a group of Jordanian university students [62]. A study by Lackaye and Margalit [89], carried out on 7th-grade and 10th-grade Israeli students, showed that there is a relationship between general feelings of academic self-efficacy and loneliness, but no such relationship was shown for self-efficacy in specific disciplines (such as history or mathematics). This may suggest that self-efficacy relates more to the general level at which a person nourishes the belief that he or she is competent and coping, rather than to very narrow areas of life.

Based on the results of our research, we were able to confirm the next hypothesis (H2a) stating that self-efficacy is positively associated with openness in adolescents’ communication with peers. This is consistent with previous findings in the field of self-efficacy and openness to communication. Prior research on adolescents has shown that young people characterized by greater self-efficacy and higher openness in communication were more likely to disclose their difficult or morally and socially problematic behavior to their mothers [90]. The study by Caprara et al. [91] investigated the role of “filial self-efficacy”, understood as adolescents’ perceived capability to communicate effectively, regulate emotions, negotiate perspectives, and exert a constructive influence within their relationships with parents. The authors found that young Italian adolescents (at the age of 15 at time 1 and 17 years old at time 2) who rated their self-efficacy in their parental relationships highly also had greater ease in maintaining open, friendly communication with their parents. Academic self-efficacy among Spanish adolescents (mean age in the first wave was 14.70; SD = 0.68) was positively correlated with peer attachment [92]. This means that the sense of being competent in a field crucial in this developmental stage, such as schooling, was accompanied by perceptions of peers as understanding of a person’s feelings and being supportive. The results of a study conducted among 12- and 13-year-olds in Turkey by Kabasakal and Emiroğlu [93] show that greater self-efficacy is associated with a higher level of acceptance of disabled peers in the classroom. A study of American adolescent girls (M = 15.2 years old; SD = 0.48) showed that social self-efficacy was positively associated with self-efficacy in sexual communication with partners and with sexual assertiveness and more frequent communication with a partner [94]. This clearly shows that confidence in one’s own ability to cope with and enter into communication allows for a more open attitude, even toward people whose reaction might be difficult to receive or who might be difficult to talk to about sensitive topics.

In line with our third hypothesis (H2b), self-efficacy was also proven to be negatively associated with difficulty in adolescents’ communication with peers. Self-efficacy, as an effect of social comparisons, allows a person to evaluate their own abilities compared to other, similar people [95]. Gaining a belief in their ability to handle, for example, relationships, supports young people in engaging in activities they believe are within their reach. Hence, people with higher self-efficacy will have less difficulty in establishing social relationships. Khademi Mofrad and Mehrabi [96] showed that self-efficacy and assertiveness are protective factors against violent behavior among Iranian adolescents (aged M = 16.39; SD = 0.49). Research by Haraldstad et al. [97] showed a negative correlation between self-efficacy and being a bully in a sample of Norwegian teenagers (12–18 years old). This may suggest that students with higher levels of self-efficacy are able to form more friendly relationships with their peers. The negative relationship between self-efficacy and difficulties in communicating with peers shown in our study is also consistent with the assumptions of self-efficacy theory regarding the role of selection processes. Highly self-efficacious individuals show a more proactive attitude in selecting and creating social environments that match their perceived abilities and resources [98]. Thereby, they may experience fewer difficulties in terms of interacting with others. In a study by Cattelino et al. [99], self-efficacy for self-regulated learning was inversely linked to depressive symptoms in adolescents. This shows that self-efficacy is a protective factor against feelings of isolation and depression, which are unfamiliar to many students at school.

Our research supports hypotheses claiming that openness in adolescents’ communication with peers (H3a) is negatively linked to loneliness, and difficulty in communication with other young people (H3b) is positively connected to loneliness. A study by Chen et al. [100] showed that among higher vocational college students (M = 20.18 years; SD = 1.96), interpersonal self-efficacy and social support are negatively associated with loneliness. In the research by Woodhouse et al. [101], shy behavior was the strongest predictor of loneliness in adolescents (11th grade students, 16–17 years), and social acceptance was the strongest protective factor against feeling alone. Similarly, in a study by Vanhalst et al. [102], among the positive predictors of loneliness in adolescents (M_age_ = 15.79 years, SD = 1.33) were shyness (which can be understood in a sense as the opposite of openness) and the experience of victimization. Negative predictors were self-esteem, feelings of social acceptance, and the quantity and quality of friendships. A review of studies conducted by González-Abaurrea et al. [103] shows a clear link between peer victimization and loneliness, which is strongest among adolescents (12–18 years old). This may be because adolescence is a time when peer relationships become central, but social and cognitive skills are not yet sufficiently developed. The results of our study join previous reports highlighting the essential role of the quality of communication with peers for young people’s perception of loneliness.

According to the mediation hypothesis (H4), openness in adolescents’ communication with peers and difficulties experienced in this area were found to be independent mediators between general self-efficacy and loneliness. This means that a strong sense of self-efficacy may predispose adolescents to a more open and approachable attitude toward peers and protect against potential problems in the field of social relations. This implies a greater ability of those convinced of their own competence to acquire social resources, which in turn reduce the risk of experiencing increased loneliness. Previous research has confirmed the mediating role of variables related to social support in the context of loneliness. Jia et al. [104] showed that social support mediates the relationship between frequency of social media use and loneliness. It should be noted that this study was conducted on a different age group (M = 44.96), but the age range (9–104) also included adolescents, and the data came from a total of 57,102 respondents. A longitudinal study conducted in a cross-lagged model by Tsai et al. [105] showed the reciprocal relationship of social self-efficacy and loneliness in Chinese students studying in the US (M_age_ = 22.92 years; SD = 3.57). The researchers noted that individuals characterized by higher social self-efficacy had more close friends among Americans. This may have promoted favorable adaptation in the new social environment and resulted in less loneliness. This is in line with Bandura’s [24] assumptions about self-efficacy. He claims that self-efficacy is a person’s own belief in his or her ability to organize and carry out the actions that are required to manage future situations. Our study demonstrated a mechanism by which we can better understand why positive beliefs about one’s own overall management abilities are linked to less loneliness. This occurs because a person is able to take care of the accessibility of other individuals, thus building interpersonal resources. Fostering young people’s sense of self-efficacy appears to be particularly important during adolescence, where feelings of a lack of social skills (as demonstrated in the group aged M = 13.34 years; SD = 2.25) [106] and vulnerability to an understated self-image and the resulting social anxiety can lead to feelings of exclusion from peer relationships (as shown in the study among people with M_age_ = 16.39; SD = 1.34) [107].

## 5. Limitations and Future Research

Among the main limitations of our research, it should be noted that adopting the assumption of mediation, although it indicates predictors, does not explain the causal relationship between the variables. In future studies, it would be valuable to address this topic using a longitudinal approach, which would allow for a more precise specification of the relationships between the variables.

The study was conducted using an Internet survey method, and this type of research does not take into account the context of experiencing loneliness. We did not consider other variables (e.g., assessment of the quality of the relationship with the parents or belonging to a group of friends) that would indicate, for example, whether the experienced loneliness is felt despite being among people or by being physically separated from them.

We also do not have knowledge of how an individual interprets the state of loneliness they experience—whether they accept it, whether they actively cope with it through hobbies, or whether they perceive loneliness only as unfavorable and unpleasant. We also included only three selected constructs. In reality, in addition to the ability to communicate openly or with difficulty, adolescents need the ability to maintain existing relationships to establish lasting and constructive bonds against loneliness.

Developing a sense of self-efficacy and the ability to communicate openly seem to be good protective resources, but the obtained results are difficult to generalize to the entire population. We do not rule out the possibility that there is a percentage of individuals who have the resources and skills to maintain open communication and yet (for example, due to environmental or socioeconomic differences) experience loneliness within their peer group.

The group was also quite homogeneous, representing teenagers studying primarily in larger cities (the regional capitals) and in general secondary schools (attended by young people aiming to pursue university education). In future studies, it would be enriching to include students from vocational schools where young people, in addition to general knowledge, develop practical skills. We are also aware that the lack of certain demographic information limits the generalizability of the results and may misrepresent the findings, e.g., regarding the level of loneliness. Therefore, further research should include information such as family type (e.g., complete/reconstructed), living in a single-family home or apartment building, the presence of immediate and extended family in the same locality, or membership in extracurricular interest groups.

It would be interesting to conduct research on a more diverse group and create a more complex model of factors that are significant for reducing loneliness in adolescents, as well as to expand the socio-demographic data. The study was conducted in Poland, and the conclusions describe to a greater extent people from Central Europe, where an intermediate type of society can be observed, between collectivist and individualistic. It would be advisable to repeat the study in a typically individualistic society (e.g., Germany) and a more collectivist one (e.g., Japan or China).

## 6. Conclusions and Implications

This study, conducted among a sample of Polish adolescents, shows that communication with peers mediates the relationship between self-efficacy and loneliness. The results suggest that adolescents who believe they are capable of achieving their goals are more likely to express their feelings in interactions with peers and exhibit fewer communication difficulties because they do not impose their opinions on others, demonstrate superiority, or exert control over others. This, in turn, enables them to establish quality relationships and reduce feelings of loneliness.

Our study demonstrates the buffering and protective role of peer communication against adverse levels of loneliness. This may inspire educational programs that support the development of communication skills in adolescents who are currently prone to reducing real-life contact with peers in favor of virtual interactions. The development of self-efficacy toward building interpersonal skills appears to be highly significant. If self-efficacy were to serve only the development of technical or professional competencies, and soft skills, including interpersonal skills, were to be neglected, there would be a risk of unbalanced development in young people. Adolescence, with its characteristic increase in social anxiety and search for identity, is a crucial period in developing a self-image as a socially competent and resourceful person. This may in turn protect against loneliness and its potential negative clinical effects.

This outcome suggests that one of the promising ways to prevent severe loneliness among adolescents is through individual and systemic work on strengthening their sense of general self-efficacy and developing communication skills, particularly in face-to-face conversations.

## Figures and Tables

**Table 1:** Descriptive statistics for self-efficacy, loneliness, openness, and difficulty in communication (N = 191).

Variables	M	SD	Skewness	Kurtosis	Shapiro–Wilk
Self-efficacy	30.30	7.10	0.104	−0.736	0.015
Loneliness	28.32	10.21	0.245	−0.944	0.001
Openness	36.47	8.09	−0.296	−0.582	0.002
Difficulty	24.72	6.34	0.033	−0.362	0.237

M—Mean; SD—Standard deviation.

**Table 2:** Pearson correlation coefficients between self-efficacy, loneliness, openness, and difficulty in communication (N = 191).

Variables	Self-Efficacy	Loneliness	Openness	Difficulty
Self-efficacy	1	−0.500 ***	0.330 ***	−0.225 ***
Loneliness		1	−0.410 ***	0.430 ***
Openness			1	−0.449 ***
Difficulty				1

*** *p* < 0.001.

**Table 3:** Role of openness in communication with peers and difficulty in communication with peers in the relationship between self-efficacy and loneliness among adolescents (N = 191).

Model	a_1_, a_2_ Paths	b_1_, b_2_ Paths	c Path	c′ Path	Indirect Effect	B (SE)	Lower CI	Upper CI
SE → OC/DC → LON	0.375 ***	−0.204 *	−0.718 ***	−0.554 ***	−0.0766	0.0382	−0.1539	−0.0055
	−0.200 **	0.436 ***			−0.0875	0.0333	−0.1603	−0.0302

*** *p* < 0.001; ** *p* < 0.01; * *p* < 0.05; SE—Self-efficacy; OC—Openness in communication with peers; DC—Difficulty in communication with peers; LON—Loneliness.

## Data Availability

Data supporting reported results can be found at https://osf.io/kjwv3/?view_only=679ac69f02b0491ea81366f8d0a5f58d (accessed on 4 August 2025).
